# A journey through the microscopic ages of DNA replication

**DOI:** 10.1007/s00709-016-1058-8

**Published:** 2016-12-09

**Authors:** Marius Reinhart, M. Cristina Cardoso

**Affiliations:** Cell Biology and Epigenetics, Department of Biology, Technische Universität Darmstadt, Schnittspahnstrasse 10, 64287 Darmstadt, Germany

**Keywords:** DNA, DNA replication, Replication foci, Replicon, Replisome, Super resolution microscopy

## Abstract

Scientific discoveries and technological advancements are inseparable but not always take place in a coherent chronological manner. In the next, we will provide a seemingly unconnected and serendipitous series of scientific facts that, in the whole, converged to unveil DNA and its duplication. We will not cover here the many and fundamental contributions from microbial genetics and in vitro biochemistry. Rather, in this journey, we will emphasize the interplay between microscopy development culminating on super resolution fluorescence microscopy (i.e., nanoscopy) and digital image analysis and its impact on our understanding of DNA duplication. We will interlace the journey with landmark concepts and experiments that have brought the cellular DNA replication field to its present state.

## The (very) early years

**Fig. 1:**
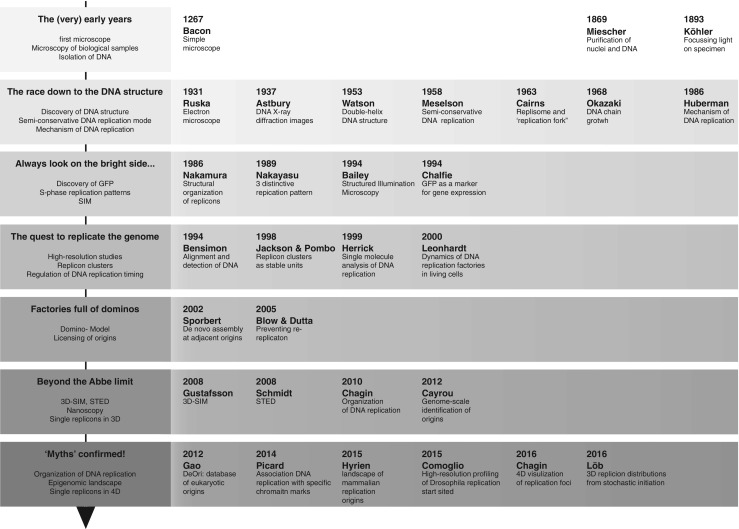
Graphical overview of microscopy developments and their impact on DNA replication studies

Long after water-filled glass bowls were used to read small letters (Singer 1914), a simple single lens microscope started the microscopic revolution (Bacon 1267) (see Table 1 and Fig. 1). Spurred throughout the ages by accidental inventions (van der Aa 1851), leaps by Galileo (Galilei 1610), and Hooke (1665), it was not until Carl Zeiss started to mass-produce microscopes in 1847 that DNA observation started to take off. Simultaneously, Mendel studied 29,000 pea plants (1866) and Haeckel postulated the containment of hereditary traits in the nucleus (1866) (Dahm 2008; Haeckel 1866), while Miescher put the microscope to good use and purified the nuclei and observed DNA (Miescher 1871). Köhler’s game-changing illumination technique (Köhler 1893) helped to perfect Zeiss UV-microscope together with Siedentopf in 1908. In 1927, shortly after Levene described the nucleic acid structure (1919), Koltsov postulated the semiconservative replication idea (Soyfer 2001).

**Table 1:** Chronological list of landmarks in microscopy and DNA replication

Year	Landmark	Author
63	Water filed glass bowls to read small letters	(Singer 1914)
1267	The first simple microscope	(Bacon 1267)
1590	Accidental discovery of the compound microscope with two (or more) lenses by Zacharias Janssen	(van der Aa 1851)
1610	“Microscope” with ×1000 magnification	(Galilei 1610)
1665	“Micrographia”	(Hooke 1665; Singer 1914)
1847	First “mass produced” microscopes in 1847	
1866	Hereditary traits in 29,000 pea plants	(Mendel 1866)
1866	Hereditary traits contained in the nucleus	(Dahm 2008; Haeckel 1866)
1871	Purified nuclei for the first time and observed DNA	(Miescher 1871)
1893	Ein neues Beleuchtungsverfahren für mikrophotographische Zwecke	(Köhler 1893)
1907	On the absorption of antibodies	
1908	First fluorescence microscopes based on UV-microscopy	
1919	Identification of the nucleic acid structure	
1927	“Replicate in a semiconservative fashion using each strand as a template”	(Soyfer 2001)
1932	Discovery of the electron microscope	(Knoll and Ruska 1932a; Knoll and Ruska 1932b)
1947	DNA X-ray diffraction images	(Astbury 1947)
1953	X-ray diffraction “Photo 51”	(Watson and Crick 1953)
1953	Discovery of the double-helix DNA structure	(Watson and Crick 1953)
1953	Discovery of phase contrast microscopy	(Zernike 1955)
1958	Confirmation of the semiconservative DNA replication model	(Meselson and Stahl 1958)
1957	Discovery of the confocal microscope	(Minsky 1961)
1962	Extraction, purification, and properties of GFP	(Shimomura et al. 1962)
1963	DNA unwinding for replication and “replication fork”	(Cairns 1963)
1966	Autoradiography of chromosomal DNA fibers from Chinese hamster cells.	(Huberman and Riggs 1966)
1966	On the mechanism of DNA replication in mammalian chromosomes	(Huberman and Riggs 1968)
1967	First practical application of the “Nipkow disk” in confocal microscopy	(Egger and Petráň 1967; Petráň et al. 1968)
1968	Mechanism of DNA chain growth. I. Possible discontinuity and unusual secondary structure of newly synthesized chains.	(Okazaki et al. 1968)
1968	Mechanism of DNA chain growth, II. Accumulation of newly synthesized short chains in *E. coli* infected with ligase-defective T4 phages.	(Sugimoto et al. 1968)
1969	Duration of the cell cycle	(Van Dilla et al. 1969)
1969	Mechanism of DNA chain growth, III. Equal annealing of T4 nascent short DNA chains with the separated complementary strands of the phage DNA	(Sugimoto et al. 1969)
1969	Mechanism of DNA chain growth. IV. Direction of synthesis of T4 short DNA chains as revealed by exonucleolytic degradation.	(Okazaki and Okazaki 1969)
1972	Bidirectional Replication of Simian Virus 40 DNA	(Danna and Nathans 1972)
1974–1979	Fork speed, replication speed, and replicon sizes	(Kriegstein and Hogness 1974; Taylor 1977; Taylor and Hozier 1976; Wilson and Wilson 1975; Yurov 1977; Yurov 1978; Yurov 1979; Yurov and Liapunova 1977)
1975	Continuous cultures of fused cells secreting antibody of predefined specificity.	
1986	Structural organizations of replicon domains during DNA synthetic phase in the mammalian nucleus	(Nakamura et al. 1986)
1989	Three distinctive replication patterns	(Nakayasu and Berezney 1989)
1992	Dynamic organization of DNA replication in mammalian cell nuclei spatially and temporally defined replication of chromosome	(O’Keefe et al. 1992)
1992	Progression of DNA synthesis	(Rizzoli et al. 1992)
1993	Structured Illumination Microscopy (SIM)	(Bailey et al. 1993)
1994	Green fluorescent protein as a marker for gene expression	(Chalfie et al. 1994)
1994	4pi microscope	(Hell 2003; Hell et al. 1994)
1994	Alignment and sensitive detection of DNA by a moving interface	(Bensimon et al. 1994)
1997	The replication origin decision point is a mitogen	(Wu and Gilbert 1997)
1997	Dynamic molecular combing: stretching the whole human genome for high-resolution studies.	(Michalet et al. 1997)
1998	Replicon clusters are stable units of chromosome structure evidence that nuclear organization contributes to the efficient activation and propagation of S phase in human cells	(Jackson and Pombo 1998)
1999	The spatial position and replication timing of chromosomal domains are both established in early G1 phase	(Dimitrova and Gilbert 1999)
1999	Single molecule analysis of DNA replication.	(Herrick and Bensimon 1999)
2000	Heterogeneity of eukaryotic replicons, replicon clusters, and replication foci	(Berezney et al. 2000)
2000	Dynamics of DNA replication factories in living cells	(Leonhardt et al. 2000)
2000	DNA replication at high resolution	(Keck and Berger 2000)
2000	Mechanisms of DNA replication	(Davey and O’Donnell 2000)
2001	Eukaryotic origins	
2001	Repression of origin assembly in metaphase depends on inhibition of RLF-BCdt1 by geminin	(Tada et al. 2001)
2001	Visualization of DNA replication on individual Epstein-Barr Virus episomes	(Norio and Schildkraut 2001)
2002	DNA polymerase clamp shows little turnover at established replication sites but sequential de novo assembly at adjacent origin clusters	(Sporbert et al. 2002)
2002	DNA replication and chromatin	(Gerbi and Bielinsky 2002)
2002	Initiation of DNA replication in multicellular eukaryotes	(Gerbi et al. 2002)
2003	Sequence-independent DNA binding and replication initiation by the human origin recognition complex	(Vashee et al. 2003)
2003	The ‘ORC cycle’: a novel pathway for regulating eukaryotic DNA replication	(DePamphilis 2003)
2004	Stable chromosomal units determine the spatial and temporal organization of DNA replication	(Sadoni et al. 2004)
2004	DNA replication and DNA repair mechanisms most of the replication machinery is also used in DNA repair.	(Sancar and Lindsey-Boltz 2004)
2005	Preventing rereplication	(Blow and Dutta 2005)
2005	PCNA acts as a stationary loading platform for transiently interacting Okazaki fragment maturation proteins	(Sporbert et al. 2005)
2005	Eukaryotic origins of DNA replication: could you please be more specific?	(Cvetic and Walter 2005)
2006	Origin selection and silent origins	(Patel et al. 2006)
2006	Regulating the licensing of DNA replication origins in metazoa	(DePamphilis et al. 2006)
2006	DNA replication: keep moving and don’t mind the gap.	(Langston and O’Donnell 2006)
2007	Impact of chromatin structure	
2007	Replisome mechanics: insights into a twin DNA polymerase machine.	(Pomerantz and O’Donnell 2007)
2007	The many faces of the origin recognition complex	(Sasaki and Gilbert 2007)
2007	High-throughput mapping of origins of replication in human cells.	(Lucas et al. 2007)
2007	Characterization of a triple DNA polymerase replisome.	(McInerney et al. 2007)
2007	Dynamic DNA helicase-DNA polymerase interactions assure processive replication fork movement.	(Hamdan et al. 2007)
2007	Polymerase switching in DNA replication.	(Lovett 2007)
2008	3D–SIM	(Gustafsson et al. 2008)
2008	Division of labor at the eukaryotic replication fork.	(Nick McElhinny et al. 2008)
2008	DNA polymerases at the replication fork in eukaryotes	(Stillman 2008)
2008	Discovery of stimulated emission depletion (STED)	(Schmidt et al. 2008)
2009	In DNA replication, the early bird catches the worm.	(Boye and Grallert 2009)
2009	G-quadruplex structures: in vivo evidence and function.	(Lipps and Rhodes 2009)
2009	Eukaryotic DNA replication control: lock and load, then fire.	(Remus and Diffley 2009)
2010	Organization of DNA replication	(Chagin et al. 2010)
2010	Eukaryotic chromosome DNA replication: where, when, and how?	(Masai et al. 2010)
2010	SCF (Cyclin F) controls centrosome homeostasis and mitotic fidelity through CP110 degradation.	(D’Angiolella et al. 2010)
2010	Uncoupling of sister replisomes during eukaryotic DNA replication.	(Yardimci et al. 2010)
2010	DNA replication: making two forks from one prereplication complex.	(Botchan and Berger 2010)
2011	Eukaryotic origin-dependent DNA replication in vitro reveals sequential action of DDK and S-CDK kinases.	(Heller et al. 2011)
2011	Failure of origin activation in response to fork stalling leads to chromosomal instability at fragile sites.	(Ozeri-Galai et al. 2011)
2011	Selective bypass of a lagging strand roadblock by the eukaryotic replicative DNA helicase.	(Fu et al. 2011)
2011	Genome-wide depletion of replication initiation events in highly transcribed regions.	(Martin et al. 2011)
2011	Origin association of Sld3, Sld7, and Cdc45 proteins is a key step for determination of origin-firing timing.	(Tanaka et al. 2011)
2012	Genome-scale identification of active DNA replication origins.	(Cayrou et al. 2012)
2012	Forkhead transcription factors establish origin timing and long-range clustering in *S. cerevisiae*	(Knott et al. 2012)
2012	A fragment based click chemistry approach towards hybrid G-quadruplex ligands: design, synthesis and biophysical evaluation	(Ritson and Moses 2012)
2012	Histone hypoacetylation is required to maintain late replication timing of constitutive heterochromatin.	(Casas-Delucchi et al. 2012)
2012	OriDB, the DNA replication origin database updated and extended.	(Siow et al. 2012)
2012	Replication timing: the early bird catches the worm.	(Douglas and Diffley 2012)
2012	CK2 inhibitor CX-4945 suppresses DNA repair response triggered by DNA-targeted anticancer drugs and augments efficacy: mechanistic rationale for drug combination therapy.	(Siddiqui-Jain et al. 2012)
2012	Experimental approaches to identify cellular G-quadruplex structures and functions.	(Di Antonio et al. 2012)
2012	Activation of the replicative DNA helicase: breaking up is hard to do.	(Boos et al. 2012)
2012	Analysis of DNA replication profiles in budding yeast and mammalian cells using DNA combing.	(Bianco et al. 2012)
2012	DeOri: a database of eukaryotic DNA replication origins.	(Gao et al. 2012)
2012	Replication origins run (ultra) deep.	(Gilbert 2012)
2012	Unraveling cell type-specific and reprogrammable human replication origin signatures associated with G-quadruplex consensus motifs.	(Besnard et al. 2012)
2012	Targeted manipulation of heterochromatin rescues MeCP2 Rett mutants and re-establishes higher order chromatin organization.	(Casas-Delucchi et al. 2012)
2013	Genome-wide mapping of human DNA-replication origins: levels of transcription at ORC1 sites regulate origin selection and replication timing.	(Dellino et al. 2013)
2013	Functional implications of genome topology.	(Cavalli and Misteli 2013)
2013	Nuclear positioning.	(Gundersen and Worman 2013)
2013	Chromatin dynamics at the replication fork: there’s more to life than histones.	(Whitehouse and Smith 2013)
2013	Quantitative, genome-wide analysis of eukaryotic replication initiation and termination.	(McGuffee et al. 2013)
2013	The Elg1 replication factor C-like complex functions in PCNA unloading during DNA replication.	(Kubota et al. 2013)
2013	Replication timing regulation of eukaryotic replicons: Rif1 as a global regulator of replication timing.	(Yamazaki et al. 2013)
2013	Bubble-seq analysis of the human genome reveals distinct chromatin-mediated mechanisms for regulating early- and late-firing origins.	(Mesner et al. 2013)
2013	A personal reflection on the replicon theory: from R1 plasmid to replication timing regulation in human cells.	(Masai 2013)
2013	From simple bacterial and archaeal replicons to replication N/U-domains.	(Hyrien et al. 2013)
2013	Genomes and G-quadruplexes: for better or for worse.	(Tarsounas and Tijsterman 2013)
2013	New insights into replication clamp unloading.	(Ulrich 2013)
2013	Replication dynamics: biases and robustness of DNA fiber analysis.	(Técher et al. 2013)
2013	Specification of DNA replication origins and genomic base composition in fission yeasts.	(Mojardín et al. 2013)
2013	The replication domain model: regulating replicon firing in the context of large-scale chromosome architecture.	(Pope and Gilbert 2013)
2013	Time to be versatile: regulation of the replication timing program in budding yeast.	(Yoshida et al. 2013)
2013	Why are there so many diverse replication machineries?	(Forterre 2013)
2014	Epigenetic control of DNA replication dynamics in mammals	(Casas-Delucchi and Cardoso 2014)
2014	Lethal effects of short-wavelength visible light on insects.	(Hori et al. 2014)
2014	Existence and consequences of G-quadruplex structures in DNA.	(Murat and Balasubramanian 2014)
2014	Histone variants: the tricksters of the chromatin world.	(Volle and Dalal 2014)
2014	Supercoiling in DNA and chromatin.	(Gilbert and Allan 2014)
2014	G4 motifs affect origin positioning and efficiency in two vertebrate replicators.	(Valton et al. 2014)
2014	The spatiotemporal program of DNA replication is associated with specific combinations of chromatin marks in human cells.	(Picard et al. 2014)
2014	Licensing of DNA replication, cancer, pluripotency and differentiation: an interlinked world?	(Champeris Tsaniras et al. 2014)
2014	Temporal and spatial regulation of eukaryotic DNA replication: from regulated initiation to genome-scale timing program.	(Renard-Guillet et al. 2014)
2014	The histone variant H2A. Bbd is enriched at sites of DNA synthesis.	(Sansoni et al. 2014)
2014	FANCJ promotes DNA synthesis through G-quadruplex structures.	(Castillo Bosch et al. 2014)
2015	The hunt for origins of DNA replication in multicellular eukaryotes.	(Urban et al. 2015)
2015	Measuring the effectiveness of scientific gatekeeping.	(Siler et al. 2015)
2015	Peaks cloaked in the mist: the landscape of mammalian replication origins.	(Hyrien 2015)
2015	Post-translational modifications of tubulin: pathways to functional diversity of microtubules.	(Song and Brady 2015)
2015	Regulated eukaryotic DNA replication origin firing with purified proteins.	(Yeeles et al. 2015)
2015	Single-molecule studies of origin licensing reveal mechanisms ensuring bidirectional helicase loading.	(Ticau et al. 2015)
2015	Single-molecule visualization of MCM2–7 DNA loading: seeing is believing.	(Chistol and Walter 2015)
2015	High-resolution profiling of Drosophila replication start sites reveals a DNA shape and chromatin signature of metazoan origins.	(Comoglio et al. 2015)
2015	The dynamics of eukaryotic replication initiation: origin specificity, licensing, and firing at the single-molecule level.	(Duzdevich et al. 2015)
2016	4D Visualization of replication foci in mammalian cells corresponding to individual replicons	(Chagin et al. 2016)
2016	3D replicon distributions arise from stochastic initiation and domino-like DNA replication progression	(Löb et al. 2016)

## The race down to the DNA structure and duplication

Phase contrast microscopy (Zernike 1955) and DNA X-ray diffraction images (Astbury 1947) Franklin, 1952, “Photo 52”) lead to fantastic images, new discoveries, and the description of the double helix DNA structure (Watson and Crick 1953). Meselson and Stahl ingeniously demonstrated the semiconservative mode of DNA replication (Meselson and Stahl 1958). The theoretical description of a confocal microscope and the first practical application of a Nipkow disk in microscopy (Egger and Petráň 1967; Petráň et al. 1968) were clear landmarks of the microscopy revolution.

Radioactive labeling and autoradiography allowed Cairns to observe DNA unwinding and the replication fork (Cairns 1963), and Huberman and Riggs observed similar replication structures in mammalian chromosomes (Huberman and Riggs 1966) and Okazaki described the lagging strand synthesis and “its” fragments (Okazaki et al. 1968; Okazaki and Okazaki 1969; Sugimoto et al. 1969; Sugimoto et al. 1968).

## Always look on the bright side

Along came *Aequorea victoria* green fluorescent protein (Shimomura et al. 1962) and brought light into darkness. Where audioradiography once ruled (Huberman and Riggs 1966; Huberman and Riggs 1968; Taylor et al. 1957), immunofluorescence labeling of fixed cells with monoclonal antibodies to modified nucleotides incorporated into newly synthesized DNA took the stage (e.g., Aten et al. 1992; Cardoso et al. 1993; Jackson and Pombo 1998; Jaunin et al. 1998; Ma et al. 1998; Mazzotti et al. 1990; Nakamura et al. 1986; Raska et al. 1989; Raska et al. 1991) only to be outshined by live cell microscopy of fluorescent fusion proteins (Cardoso et al. 1997; Leonhardt et al. 2000). Cell cycle duration (Van Dilla et al. 1969), fork speed, replication rate, and replicon sizes (Kriegstein and Hogness 1974; Taylor 1977; Taylor and Hozier 1976; Wilson and Wilson 1975; Yurov 1977; Yurov 1978; Yurov 1979; Yurov and Liapunova 1977) were all unearthed from the dark.

In parallel, the first affordable home computers made digital image analysis possible through the help of Wayne S. Rasband who developed the milestone in image analysis ImageJ (then, NIH Image) in 1987 (Schneider et al. 2012).

**Fig. 2:**
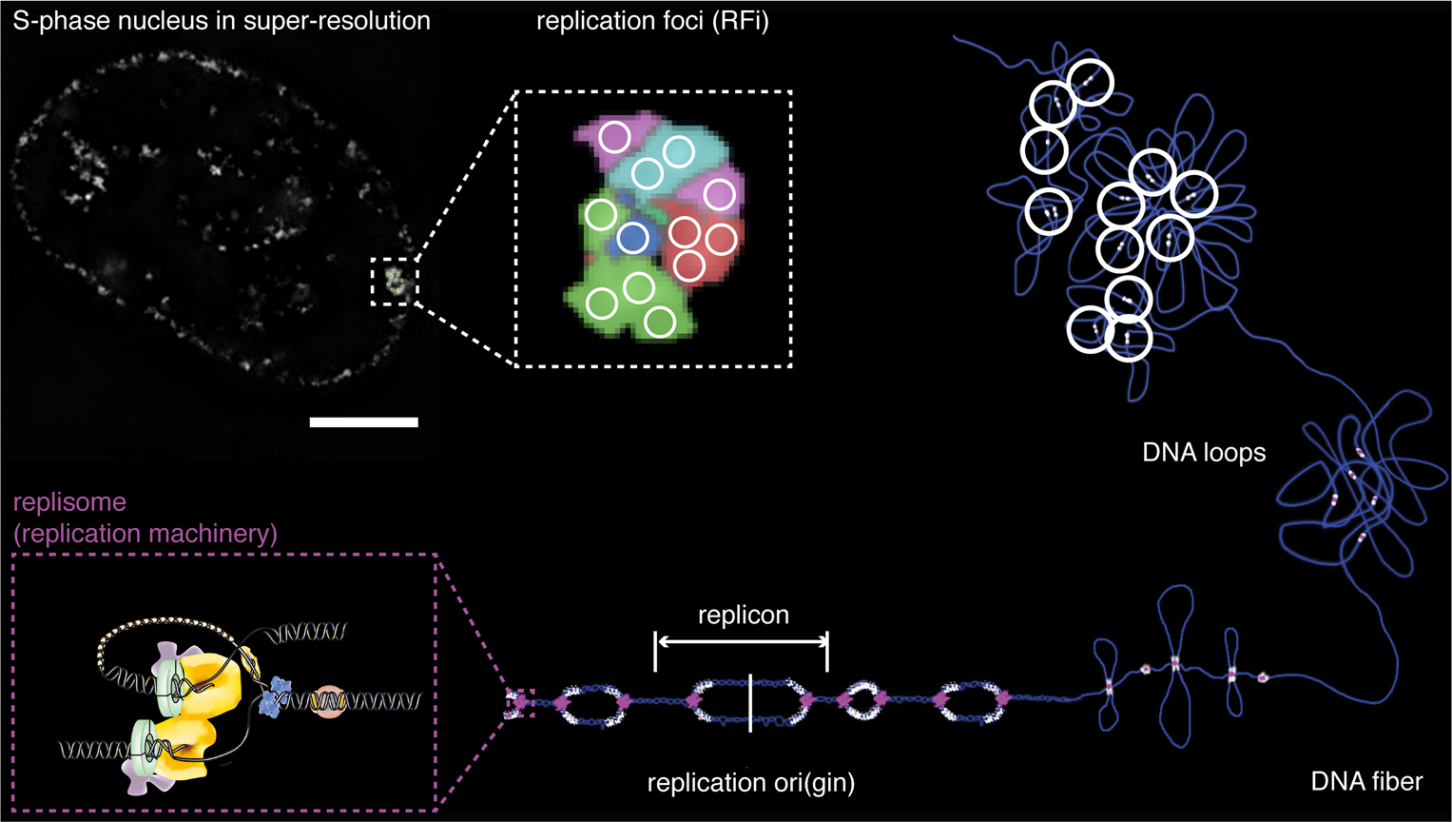
Organization of DNA replication from the genome to the individual replisome/replicon. A fluorescently labeled human HeLa Kyoto cell with a typical late S-phase replication pattern is presented in the top left corner (*scale bar* = 5 μm). Magnified super-resolution replication foci, with *white circles* representing individual replication sites displayed in the *middle of the top row*. A scheme of clustered DNA loops with active replication sites (*white*) is shown on the *right*. Starting point of DNA replication, the replication origin (ori), and the region replicated from a single origin displayed in the *bottom row*. Each replicon is replicated by two replication machineries (*magenta*), composed of various replication proteins, magnified in the *bottom left corner*. Adapted from (Chagin et al. 2016; Chagin et al. 2010)

Extensive microscopic analysis in fixed cells followed and provided a spatiotemporal description of replication sites (replication foci; see Fig. 2) in cells throughout S-phase (Nakamura et al. 1986) along with the three main distinctive early, mid, and late S-phase replication foci patterns (Jackson and Pombo 1998; Mills et al. 1989; Nakayasu and Berezney 1989). Alongside, replication origins (Burhans et al. 1990; Burhans et al. 1991) were also reported.

## The quest to replicate the genome

DNA loops (see Fig. 2) and their “functional” attachments to active transcription units were shown as chromatin organizers during mitosis (Jackson et al. 1992), and replication factories were proposed as clusters of DNA replication sites organized by the nucleoskeleton (Hozák et al. 1993). Molecular combing, refined DNA fiber analysis, and sensitive detection of DNA (Bensimon et al. 1994) opened the door to whole genome stretching and high-resolution studies (Michalet et al. 1997). It allowed analysis of single DNA molecules undergoing replication (see Fig. 2) in a much greater resolution (Herrick and Bensimon 1999) than ever before. Stable replicon clusters were also described as a basis for effective activation and propagation of genome replication during S-phase (Jackson and Pombo 1998) and regulation of replication timing (Dimitrova and Gilbert 1999).

Studies on DNA replication proteins (see Fig. 2) using live-cell fluorescence microscopy produced time lapse movies of replication factories and elucidated basic principles of their dynamic assembly-disassembly behavior (Leonhardt et al. 2000). Different regulatory levels were shown to be necessary to initiate and regulate DNA replication. Not only the chromatin structure, nuclear, and chromosomal locations but also origin recognition complex (ORC) and a whole bunch of other factors were found to define start sites of replication (DePamphilis 2003; Gerbi and Bielinsky 2002; Gerbi et al. 2002; Sasaki and Gilbert 2007).

## Factories full of dominos

In addition to the “factory model” (Hozák et al. 1993), more dynamic models ensued (Sadoni et al. 2004; Sporbert et al. 2002) whereby replication at one site induces domino-like activation of neighboring origins, without the need to postulate pre-determined clusters of replicons. The combination with an earlier model postulating that origins of replication would be licensed only during mitosis and this license to replicate would be revoked after one round of replication (Blow and Dutta 2005; Blow and Laskey 1988) elegantly demonstrated how DNA is completely duplicated once, and only once, during each cell cycle. Despite Cvetic wishing for “eukaryotic origins of DNA replication to please be more specific” (Cvetic and Walter 2005), DNA replication origins in higher eukaryotes have been at best elusive. Nonetheless, as a whole, DNA replication is a very robust mechanism and stalled forks can be reactivated or reactivate neighboring origins to close all gaps and provide a perfect copy of billions of nucleotides at every cell division (Langston and O’Donnell 2006; Patel et al. 2006).

## The ever elusive origin

The hunt for the elusive consensus motif of DNA replication origins continued with genome-wide high throughput mapping of potential origins and next-generation sequencing methods (Besnard et al. 2012; Cadoret et al. 2008; Cayrou et al. 2012; Dellino et al. 2013; Karnani et al. 2010; Lucas et al. 2007; Martin et al. 2011; Mesner et al. 2013; Mesner et al. 2011; Mukhopadhyay et al. 2014; Picard et al. 2014; Valenzuela et al. 2011) but stalled without a conclusive definition of the mammalian origin of replication. Correlations with specialized DNA structures (e.g., G-quadruplexes) and many others have been suggested but there seems not to be a simple solution and potentially there is no need to have one.

Studies into the epigenomic landscape, epigenetic control of DNA replication, and higher order chromatin organization (Casas-Delucchi and Cardoso 2011; Casas-Delucchi et al. 2012) have provided a link of epigenetic modifications (in particular, histone acetylation level) and temporal control of DNA replication origin firing.

Altogether, even Hyrien’s “Peaks cloaked in the mist,” all out approach was not able to identify possible origins by similarities in thousands of microarrays and/or next-generation sequencing data, suggesting origins form at unspecific DNA sites, but are suppressed by ongoing transcription (Hyrien 2015), which is highly correlated with histone acetylation.

## To go where no one has gone before: beyond the Abbe limit

Meanwhile, the microscopy arms race to and beyond the diffraction limit calculated by Abbe continued with the Structured Illumination Microscopy (SIM) (Bailey et al. 1993), the 3D–SIM (Gustafsson et al. 2008) and the stimulated emission depletion (STED) (Schmidt et al. 2008).

**Fig. 3:**
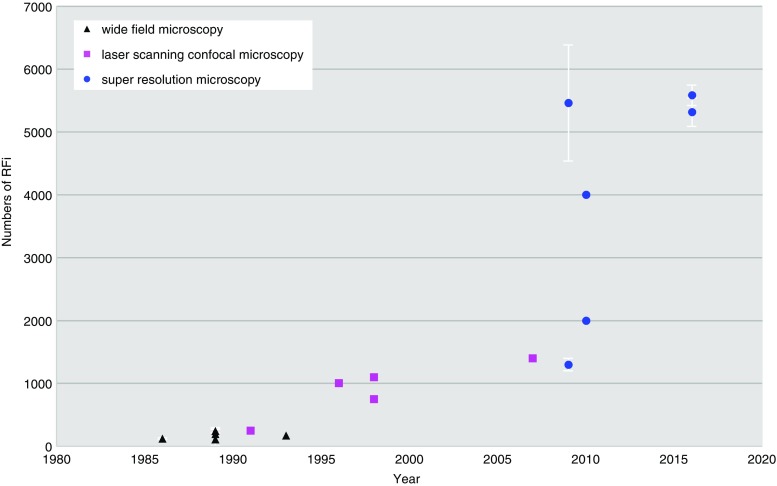
Graphical overview of replication foci numbers in correlation with microscopy developments

The first attempts to label dating back to 1986 (Nakamura et al. 1986) and quantify replication sites in cells yielded numbers on the low hundreds (see Fig. 3). A decade later with the advent of digital imaging and computational image analysis tools, these numbers grew to around one thousand (Berezney et al. 1996; Fox et al. 1991; Jackson and Pombo 1998; Ma et al. 1998), where they remained for several years (see Fig. 3). Such numbers of replication sites were compatible with a concept of clusters of replicons activated together and, thus, visualized together.

The dramatic increase in spatial resolution made possible with the new super-resolution microscopy techniques (fluorescence nanoscopy) enabled the visualization, for the first time, of smaller replication structures (Baddeley et al. 2010; Cseresnyes et al. 2009; Ligasová et al. 2009). It was now possible to resolve structures well below the Abbe limit, down to 30 nm and smaller. Nanoscopy (Gustafsson et al. 2008; Hell 2003; Hell et al. 1994) is in full swing and let us go where no one has gone before: beyond the Abbe limit. This, on the other hand, created another level of demand upon image analysis tools.

## “Myths” confirmed!

The stage was now set to try and unveil the units of genome replication, i.e., the replicons and their associated machinery, the replisome, in cells.

From the earlier studies using light nanoscopy techniques (Baddeley et al. 2010; Cseresnyes et al. 2009) as well as electron microscopy (Koberna et al. 2005), suitable computational image analysis protocols were developed (Chagin et al. 2015). These combined efforts led to a further increase in the numbers of replication sites measured in cells (see Fig. 3), which was now finally compatible and fitting with the predicted numbers of replicons needed to duplicate the genome in human cells (Chagin et al. 2016; Löb et al. 2016).

The microscopic information age had arrived. Previous efforts by Shaw et al. (2010), together with measurements throughout the years culminating on the visualization and quantification of individual replicons in cells in 4D, all supported by 3D–SIM imaging (Chagin et al. 2016) were all combined in a minimalistic but comprehensive 4D replicon simulation model (Löb et al. 2016) displaying previously published replication polarity gradients, replication timing profiles, N/U domains, topologically associating domains, and timing transition regions (Audit et al. 2013; Baker et al. 2012; Chen et al. 2010; Hyrien et al. 2013; Pope et al. 2014).

## Journey into the future

Future work should aim to bridge the ever-increasing genome-wide population data, with single molecule and single-cell microscopic data. Novel ways to combine and relate these very different types of information should be developed to get the highest spatial together with the highest temporal resolution without compromising the data on variability between single cells.

Importantly, the available models should be put to work to predict and test genome replication in different cell types and species and under different stress conditions. This would unleash the value of the existing models and lead us into the in silico DNA replication era.
